# Profile of the nursing diagnoses in stable heart disease patients

**DOI:** 10.17533/udea.iee.v37n2e08

**Published:** 2019-09-19

**Authors:** Patrícia Cristina Cardoso¹, Larissa Gussatschenko Caballero², Karen Brasil Ruschel³, Maria Antonieta Pereira de Moraes, Eneida Rejane Rabello da Silva

**Affiliations:** 1 Registered Nurse, Ph.D. candidate. Clinical Hospital of Porto Alegre, Brazil. Email: patricia.cardoso@acad.pucrs.br Clinical Hospital of Porto Alegre Brazil patricia.cardoso@acad.pucrs.br; 2 Registered Nurse, MSc. Clinical Hospital of Porto Alegre, Brazil. Email: lcaballero@hcpa.edu.br Clinical Hospital of Porto Alegre Brazil lcaballero@hcpa.edu.br; 3 Registered Nurse, PhD. Researcher, Federal University of Rio Grande do Sul; Institute for Health Technology Assessment, Brazil. Email: karenbruschel@gmail.com Universidade Federal do Rio Grande do Sul Federal University of Rio Grande do Sul Brazil karenbruschel@gmail.com; 4 Registered Nurse, PhD. Rio Grande do Sul Cardiology Institute/University Foundation of Cardiology, Brazil. Email: moraes.enf@cardiologia.org.br Rio Grande do Sul Cardiology Institute/University Foundation of Cardiology Brazil moraes.enf@cardiologia.org.br; 5 Registered Nurse, PhD. Associate professor, Federal University of Rio Grande do Sul, Brazil. Email: eneidarabelo@gmail.com Universidade Federal do Rio Grande do Sul Federal University of Rio Grande do Sul Brazil eneidarabelo@gmail.com

**Keywords:** ambulatory care, cross-sectional studies, nursing diagnosis, outpatients, nursing process, myocardial ischemia., atención ambulatoria, diagnóstico de enfermería, estudios transversales, pacientes ambulatorios, proceso de enfermería, isquemia miocárdica.

## Abstract

**Objective.:**

To identify the nursing diagnoses through reports in the medical records of patients monitored in a specialized ischemic heart disease outpatient clinic.

**Methods.:**

Cross-sectional study with retrospective data collection in the medical records. From the data collected, the nursing diagnoses were proposed by the researchers and submitted for validation by specialist cardiology nurses.

**Results.:**

A total of 13 nursing diagnoses were evaluated from the medical records of 50 outpatients with the following validation agreements among the specialists: Ineffective health management (100%), Noncompliance (100%), Sedentary lifestyle (100%), Activity intolerance (100%), Decreased cardiac output (88%), Risk of decreased cardiac tissue perfusion (65%), Risk of intolerance to activity (65%), Acute pain (76%), Ineffective health maintenance (65%), Risk-prone health behavior (65%), Risk for decreased cardiac output (65%), Risk for intolerance to activity (65%), Ineffective respiratory pattern (53%), Impaired memory (29%).

**Conclusion.:**

In this study, the nursing diagnoses validated for stable heart disease patients were linked to adherence to treatment and to the cardiovascular responses of the patients, reinforcing the importance of early intervention. These results allow the multidisciplinary team to individualize the goals and interventions proposed for ischemic heart disease patients.

## Resumen

**Objetivo.:**

Identificar los diagnósticos de enfermería a partir de los registros en el seguimiento de los pacientes que acuden a un centro ambulatorio especializado en cardiopatía isquémica en la ciudad de Porto Alegre, Brasil.

**Métodos.:**

Estudio transversal con toma de información retrospectiva de la historia clínica. A partir de los datos recolectados, los diagnósticos de enfermería identificados por los investigadores se sometieron a valoración de enfermeros especialistas en cardiología.

**Resultados.:**

Se identificaron 13 diagnósticos de enfermería en las historias clínicas de 50 pacientes ambulatorios, con la siguiente concordancia de validación entre los especialistas: control ineficaz de la salud (100%), falta de adherencia (100%), estilo de vida sedentario (100%), Intolerancia a la actividad (100%), Débito cardíaco disminuido (88%), Riego de perfusión tisular cardíaca disminuida (76%), Dolor agudo (76%), Mantenimiento ineficaz de la salud (65%), Comportamiento de salud propenso a riesgo (65%), Riesgo de débito cardíaco disminuido (65%), Riesgo de intolerancia a la actividad (65%), Patrón respiratorio ineficaz (53%), Memoria perjudicada (29%).

**Conclusión.:**

En este estudio los diagnósticos de enfermería validados para los pacientes cardiópatas estables están relacionados con la adherencia al tratamiento y a la respuesta cardiovascular a las intervenciones, reforzando la importancia de intervención precoz. Esos resultados permiten, en equipos multiprofesionales, individualizar las metas e intervenciones para los pacientes con cardiopatía isquémica.

## Introduction

Despite cultural changes, increased life expectancy and increasing technological advances, the incidence of cardiovascular diseases is still significant in the current society, accounting for more than 17.9 million deaths worldwide in 2016.(1) In Brazil, mortality due to cardiovascular diseases represented 28% of all deaths in the previous five years.(2) The classic study ‘The global study of risk factors for acute myocardial infarction’ (INTER-HEART) evaluated cardiovascular risk factors in 52 countries. It was found that nine risk factors, which are simple to detect and susceptible to change, account for more than 90% of the attributable risk of cardiovascular diseases. Of these, six potentiate the risk of clinical events (dyslipidemia, hypertension, diabetes mellitus, overweight/obesity, smoking, and psychological stress) and three collaborate to reduce this risk (regular exercise, adequate consumption of vegetables and fruits, and small to moderate doses of alcohol).(3) 

The study ‘Brazilian Intervention to Increase Evidence Usage in Acute Coronary Syndromes’ (BRIDGE-ACS), a multi-centric training program initiative to increase the use of evidence-based therapies, quantified an improvement in the quality of the therapy provided by the implementation of very simple educational measures, which can be implemented at low cost.(4) The analysis of chronic heart disease patients, as in a recently published randomized clinical trial, demonstrates this situation in the clinical practice by proving that systematic guidance approaches combined with telephone reinforcement are more effective than standard clinical monitoring consultations.(5) The multiplicity of factors that influence the health conditions of individuals with coronary artery disease justifies the importance of an extended and effective care practice to fulfill the needs of these patients.(6) The application and systematization of care through the Nursing Process steps has been shown to be a scientific method that favors the clinical judgment of the nurse. The use of the nursing process together with a standardized language system, such as that of NANDA International, Inc (NANDA-I) allows nurses to determine the most accurate nursing diagnoses for an individual, community or family. Its use contributes to patient safety by reducing dubious and undue information and allowing appropriate interventions to be selected.(7) 

The nursing diagnosis is part of the nursing process and results from the clinical judgment of information obtained from the nursing consultation.(7) The nursing diagnosis is indispensable for the nursing process, since it is the basis for planning interventions and for the nursing outcomes.(8) Accordingly, nurses can act based on evidence, identifying human responses and establishing strategies for health recovery and/or the improvement of the individual or collective well-being.(9) Establishing nursing diagnoses provides innumerable benefits, such as accurately and quickly understanding the patient’s needs, facilitating the continuity of care using standardized language, guiding the choice of nursing interventions that allow better results, determining care priorities, qualifying care, and promoting the development of the profession.(10,11) From this perspective, this study aimed to identify nursing diagnoses from signs and symptoms (defining characteristics), clues or clinical evidence in patients in outpatient monitoring with stable Ischemic Heart Disease, according to NANDA-I. This study is relevant for the practice as it equips the nursing team for an accurate clinical evaluation, with the planning of the expected results and interventions proposed in order to maximize teamwork. 

## Methods

This was a cross-sectional study with retrospective data collection in the electronic medical records, conducted in an ischemic heart disease outpatient clinic of a University Hospital in the city of Porto Alegre, Brazil. In this outpatient clinic, 50 patients were being monitored. At the first outpatient visit, the following evaluations are performed: anthropometric data collection, clinical examination, and identification of risk factors, the social and family context and the patients’ life habits. From these data, the team plans goals for modifications of the lifestyle of the patient, and gives guidance regarding adherence to the treatment. For monitoring in this outpatient clinic, patients must present two or more of the following inclusion criteria: Blood Pressure > 140x90 mmHg with the use of antihypertensives; Body Mass Index > 25 kg/m^2^; glycated hemoglobin > 6.5%; sedentary lifestyle; currently smoking; Low-density lipoprotein > 150 mg/dl with the use of statins; and triglycerides > 150 mg/dl. For this study all 50 patients referred to the team had their records evaluated.

Data collection was performed by two researchers in January and February 2016, through the reading of the electronic medical records, evaluating the record of the first consultation performed by the outpatient clinic staff. The interval of interest was defined from the inauguration of the outpatient clinic (April 2014) to the month immediately prior to the collection of the information (December 2015). Patients whose medical records did not present a record of evolution in the outpatient clinic of ischemic heart disease during the period of interest were excluded from the study.

The information was transcribed to a structured form with two sections: patient identification, which included data such as name, age, sex, color and comorbidities; and anamnesis and physical examination data. From these data, in addition to clinical experience and support from the literature, thirteen probable NANDA-I nursing diagnoses were pre-selected based on the defining characteristics, risk factors, related factors, clues, signs and/or possible symptoms. The selected diagnoses were transcribed to a form according to NANDA-I taxonomy(6) and submitted for evaluation and validation by 17 specialist nurses - all with specialization or Master’s degrees in cardiology and over five years of experience in the clinical practice. For this step a document was drawn up listing the diagnoses identified for the study patients, as well as the defining characteristics, related factors or risk factors, with the respective prevalences. The document was printed and delivered personally to the specialists, to carry out the analysis of the content and the judgment of agreement or not with the validation of the proposed nursing diagnoses. The participants had 15 days to complete this instrument and return it to the researchers. After completion of the document and signing of the consent form, the document was delivered directly to the researchers at an agreed time and place. Only the nursing diagnoses that were selected by 100% of the specialists were considered valid. The choice of this percentage was based on similar studies that considered a consensus of 100% among the specialists in order to confer greater consistency, solidity and applicability on the inferred diagnoses.(12)

The data were analyzed using the Statistical Package for the Social Sciences, version 21.0 program. Continuous variables were expressed as mean and standard deviation, median and interquartile range. Categorical variables were presented with absolute numbers and percentages. This study was approved by the research ethics committee of the institution.

## Results

The 50 patients being monitored by the team at the outpatient clinic were included, with no exclusions due to inadequate registration. Of the 50 patient medical records, 64% were from males, mean age 64 (± 7.7) years, white (82%), married (66%), working (62%), with incomplete elementary education (54%) and living with family members (64%). Regarding the clinical characteristics, the patients presented systemic hypertension (96%), diabetes mellitus (48%), acute coronary syndrome (66%), coronary artery bypass grafting (22%) and heart failure (16%). The other data referring to the characteristics of the group of participants are described in Table 1.

**Table 1:** Sociodemographic and clinical characteristics of 50 stable heat disease patients

Variable	n=50
Sociodemographic characteristic	
Age; Mean ± standard deviation	64±7.7
Male; n (%)	32 (64)
White color; n (%)	41 (82)
Marital status: married; n (%)	33 (66)
Occupation: working; n (%)	31 (62)
Schooling: incomplete fundamental education; n (%)	27 (54)
Lives with family members; n (%)	32 (64)
Clinical Characteristics	
Sedentary lifestyle; n (%)	31 (62)
Smoking; n (%)	14 (28)
Body mass index; Mean ± standard deviation	30±5.0
Systemic hypertension; n (%)	48 (96)
Type II Diabetes Mellitus; n (%)	24 (48)
Percutaneous Transluminal Coronary Angioplasty;n (%)	38 (76)
Acute Coronary Syndrome; n (%)	33 (66)
Coronary Artery Bypass Grafting; n (%)	11 (22)
Cardiac Insufficiency; n (%)	8 (16)
Glycated hemoglobin; Mean ± standard deviation	9.3±3.0
Triglycerides mg/dl; Median and interquartile range	208 (120-355)
High density lipoprotein mg/dl; Mean ± standard deviation	36.1±9.2
Low density lipoprotein mg/dl; Median and interquartile range	97 (68.5-162)
Total cholesterol mg/dl; Mean ± standard deviation	187±59.0

During the review of the medical records, the predominant defining characteristics were: difficulty with the prescribed regimen 49 (98%), choices in the daily life ineffective to achieve health goals 49 (98%), failure to act in order to prevent problems (88%), lack of adherence behavior 44 (88%), failure to act to reduce risk factors 43 (86%), and failure to include the treatment regimen in the daily life 42 (84%). The related factors most identified in the records were: insufficient knowledge of the therapeutic regimen 49 (98%), complex treatment regimen 46 (92%), inadequate number of indications of action 43 (86%), prolonged duration of the regimen 43 (86%), failure to achieve results 42 (84%), and low self-efficacy 42 (84%). From this investigation, 13 NDs were proposed for validation by the specialist nurses, with the respective NANDA-I taxonomy coding and the percentage of agreement among the specialists, as presented in Table 2.

**Table 2:** Nursing diagnoses evaluated by the 17 specialists and their respective agreement

Nursing diagnosis	n (%)
Ineffective health management (00078)	17 (100)
Noncompliance (00079)	17 (100)
Sedentary lifestyle (00168)	17 (100)
Activity intolerance (00092)	17 (100)
Decreased cardiac output (00029)	15 (88)
Risk for decreased cardiac tissue perfusion (00200)	13 (76)
Acute pain (00132)	13 (76)
Ineffective health maintenance (00099)	11 (65)
Risk-prone health behavior (00188)	11 (65)
Risk for decreased cardiac output (00240)	11 (65)
Risk for activity intolerance (00094)	11 (65)
Ineffective respiratory pattern (00032)	9 (53)
Impaired memory (00131)	5 (29)

At the end of the validation process by the specialists, four NDs were indicated with 100% agreement, according to table 3: Sedentary lifestyle (00168), Ineffective health management (00078), Noncompliance (00079) and Activity intolerance 00092). The first three are located in the Health Promotion domain, while the final one is in the Activity/Rest domain. The respective classes to which these NDs belong, within each domain, are class 1 (Health Awareness); class 2 (Health Management); class 2 (Health Management); and class 4 (Cardiovascular-pulmonary responses).

**Table 3:** Nursing diagnoses validated by the 17 specialists in 50 stable cardiac patients

Nursing diagnosis	Patients n (%)
Sedentary Lifestyle (00168)	
Defining characteristics	
Daily physical activity less than recommended for gender and age	17 (34)
Lack of physical fitness	15 (30)
Related factors	
Insufficient interest in physical activity	10 (20)
Insufficient motivation for physical activity	14 (28)
Insufficient training	1 (2)
Ineffective health management (00078)	
Defining characteristics	
Difficulty with prescribed regimen	46 (92)
Ineffective daily choices to achieve health goals	43 (86)
Failure to act to reduce risk factors	44 (88)
Failure to include the regimen of treatment into daily life	43 (86)
Related factors	
Family conflict	1 (2)
Insufficient knowledge of the therapeutic regimen	10 (20)
Inadequate number of indications of action	43 (86)
Complex treatment regime	46 (92)
Noncompliance (00079)	
Defining characteristics	
Noncompliance behavior	44 (88)
Exacerbation of symptoms	22 (44)
Failure to achieve results	42 (84)
Related factors	
Prolonged duration of the regime	43 (86)
Complex treatment regime	43 (86)
Activity intolerance (00092)	
Defining characteristics	
Discomfort in efforts	25 (50)
Dyspnea on exertion	12 (24)
Fatigue	5 (10)
Related factors	
Imbalance between oxygen supply and demand	24 (48)
Sedentary lifestyle	02 (04)
Insufficient knowledge of the regime	17 (34)

## Discussion

This is the first study that proposed to study the records of an outpatient team specialized in Ischemic Heart Disease, aiming to establish the potential nursing diagnoses for this population. The analysis of the records allowed specialist nurses in the area of cardiology to validate four nursing diagnoses for outpatients with ischemic heart disease: Ineffective health management (00078); Noncompliance (00079); Sedentary lifestyle (00168) and Activity intolerance (00092). When analyzing the domains and classes of the groupings established by the NANDA-I, it was verified that three of the four validated nursing diagnoses are related to life habits. Considering the breadth and complexity of the lifestyle problem, especially in the context of patients with ischemic heart disease, understanding the determinant factors favors an expanded view of the causes and consequences of an unhealthy lifestyle.(13) Together with adherence to lifestyle changes, regular use of medications is also a challenge for health staff. About 50% of patients with ischemic heart disease have poor adherence to treatment, and consequently, no clinical benefit is obtained from the use of medications.(14) Failure to comply with the established therapeutic goals is related to a greater number of hospitalizations, higher costs of the disease and worse quality of life, since the complications are installed earlier and more intensely.(15) Accordingly, team approaches can favor the understanding of the patients, improve the bond with family members and caregivers, and provide greater adherence to treatment. 

Considering the Noncompliance (00079) and Ineffective health management (00078) nursing diagnoses, both contained in the Health Management class of the Health Promotion domain, the importance of addressing this complex issue is reinforced, as is the difficulty in modifying behavior. Adherence, according to the 1997 report of the American Heart Association, has been understood as a behavioral process, heavily influenced by the environment in which the patient lives, including health practices and systems - an assumption that the patient possesses the knowledge, motivation, skills and resources necessary to follow the recommendations of the healthcare provider.(16) Currently, adherence is understood as a more comprehensive concept, influenced by several factors related to the patient, the complexity of the treatment, the health services, and the provider-user relationship. In this way, it is understood that the patient is not the only one responsible for their treatment.(17,18) In this scenario, patient satisfaction was referenced as being fundamental for adherence. Satisfaction levels are related to several components of the consultation, namely affective aspects (emotional support and comprehension), behavioral aspects (adequate prescriptions and explanations) and aspects related to the competence of the health professional (appropriate diagnosis, treatment and referral), as well as the content of the consultations provided, in which the patients should receive as much information as possible, conveyed in the best way.(19)

It is recognized that the need for greater changes in habits or lifestyle due to the treatment (cessation of smoking and alcohol intake, dietary restrictions and the performance of physical activity) reduces the chances of more adherence.(19) The difficulty of incorporating routine physical exercises, indicated by the Sedentary lifestyle (00168) diagnosis, should take into account the repercussion of this practice on the health status of the individual. Evaluating the possible and probable impacts on patients’ daily lives implies broadening the care routine to design goals to be agreed with the patient based on the knowledge of their attitudes, beliefs and health habits - since they explain the reason for sedentary behaviors rather than the practice of physical exercise.(10) In this specific group of patients, the related factors that help confirm this nursing diagnosis are Insufficient interest in physical activity and Insufficient motivation for physical activity, which, if their percentages are added, were present in almost half of the sample [24 (48%)]. On the other hand, the Activity intolerance (00092) nursing diagnosis presented, among the defining characteristics and risk factors that interfere in the practice of physical activity, dyspnea on exertion [12 (24%)], fatigue [5 (10%)] imbalance between oxygen supply and demand [24 (48%)], stress discomfort [25 (50%)] and the presence of angina [37 (74%)]. These factors are recognized as restrictive for the performance of physical practices, especially activities with aerobic increase. In addition, stress discomfort seems to be one of the main limitations to physical exercise, not only due to the indication of angina, but also due to the association that many chronic patients (especially our population, in which more than half, 66%, presented acute coronary syndrome) make between this pain and a condition of a major adverse clinical event - emphasizing a potential psychological restriction for the full practice of physical activities.

Despite decades of study and various models and theories of adherence it has not yet been possible to come up with an effective answer to resolve this issue. Risk factors for coronary artery disease, as well as other diseases, coupled with the need for a complex therapeutic regimen, make it difficult for patients to fully adhere, especially considering changes that should become more of a healthy lifestyle rather than a habit.

The limitations of the present study are due to its retrospective nature and the search in medical records.

The results of this study allowed four diagnoses to be validated for patients with stable heart disease, including Ineffective health management (00078); Noncompliance (00079); Sedentary lifestyle (00168); and Activity Intolerance (00092). These diagnoses are linked to adherence to the treatment and to the cardiovascular responses of the patients, reinforcing the importance of early intervention. This information allows the context of the patient to be identified and, therefore, the care and evaluation to be planned, with a view to the rehabilitation and prevention of the progression of the disease. In this context, the establishment of nursing diagnoses is considered the guiding factor for choosing the most appropriate interventions to achieve the results expected for each individual. 
